# Association of Neighborhood Economic Trajectories With Changes in Weight Status Among Black and White Adults in the Southeastern US

**DOI:** 10.1001/jamanetworkopen.2022.30697

**Published:** 2022-09-08

**Authors:** Qian Xiao, Eric Myott, David G. Schlundt, William Stancil

**Affiliations:** 1 Department of Epidemiology, Human Genetics, and Environmental Sciences, University of Texas Health Science Center at Houston School of Public Health, Houston; 2 Institute on Metropolitan Opportunity, University of Minnesota, Minneapolis; 3 Department of Psychology, Vanderbilt University, Nashville, Tennessee

## Abstract

**Importance:**

Neighborhood environment is an important factor associated with population disparities in obesity. However, few studies have examined whether and in what ways long-term trajectories of neighborhood conditions may be associated with weight outcomes. Moreover, there is a lack of research focusing on multidimensional and nuanced measures that make distinctions between multiple types of neighborhood change (eg, gentrification vs overall growth).

**Objective:**

To examine the association between long-term neighborhood economic trajectories and changes in weight status among Black and White adults residing in predominantly low-income communities in the southeastern US.

**Design, Setting, and Participants:**

This study was a longitudinal analysis of participants in the Southern Community Cohort Study. Five types of neighborhood economic trajectories (stability, growth, displacement, abandonment, and poverty concentration) were measured using data from the US Census and the American Community Survey from 2000 to 2016. Data were analyzed from December 12, 2021, to July 16, 2022. A total of 33 621 Black and White adults in the southeastern US were included in the analytic sample.

**Exposure:**

Neighborhood economic trajectory.

**Main Outcomes and Measures:**

Substantial weight gain and substantial weight loss (ie, gaining or losing ≥10% of baseline weight) between baseline (March 2002 to September 2009) and follow-up (November 2008 to January 2013) periods were assessed using self-reported information.

**Results:**

Among 33 621 participants, the mean (SD) age was 53.4 (8.8) years; 22 116 participants (65.8%) were women, 21 782 (64.8%) were Black, and 11 839 (35.2%) were White. Compared with residents in neighborhoods with stable trajectories, those in neighborhoods with growth trajectories that did not displace original residents were less likely to experience substantial weight gain (odds ratio [OR], 0.75; 95% CI, 0.58-0.97), whereas those in neighborhoods with poverty concentration trajectories were more likely to experience substantial weight gain (OR, 1.08; 95% CI, 1.00-1.17). These patterns appeared stronger among Black participants (eg, substantial weight gain in poverty concentration group: OR, 1.10 [95% CI, 1.00-1.22]; in growth group: OR, 0.76 [95% CI, 0.56-1.02]) compared with White participants (eg, substantial weight gain in poverty concentration group: OR, 1.03 [95% CI, 0.90-1.18]; in growth group: OR, 0.84 [95% CI, 0.52-1.36]). Differences in patterns were also observed among men (eg, substantial weight gain in poverty concentration group: OR, 1.02 [95% CI, 0.88-1.17]; in growth group: OR, 0.58 [95% CI, 0.35-0.96]) compared with women (eg, substantial weight gain in poverty concentration group: OR, 1.12 [95% CI, 1.02-1.23]; in growth group: OR, 0.83 [95% CI, 0.62-1.12]). However, none of the interaction terms between Black vs White participants and men vs women were statistically significant. Neighborhood trajectory was not associated with substantial weight loss (poverty concentration group: OR, 1.00 [95% CI, 0.93-1.09]; abandonment group: OR, 1.01 [95% CI, 0.84-1.15]; displacement group: OR, 1.04 [95% CI, 0.83-1.23]; growth group: OR, 0.88 [95% CI, 0.69-1.12]).

**Conclusions and Relevance:**

In this cohort study, neighborhood economic trajectories were associated with weight gain. These findings highlight the importance of using more nuanced and multidimensional measures of neighborhood change in public health research.

## Key Points

**Question:**

Are long-term neighborhood economic trajectories associated with weight gain and loss among Black and White adults in the southeastern US?

**Findings:**

In this cohort study of 33 621 Black and White adults from predominantly low-income communities in the southeastern US, different neighborhood economic trajectories were associated with weight gain. Compared with residents in neighborhoods with stable economic trajectories, those in neighborhoods with economic growth trajectories were less likely to experience substantial weight gain, whereas those in neighborhoods with poverty concentration trajectories were more likely to experience substantial weight gain.

**Meaning:**

This study’s findings suggest that neighborhood change plays a role in population health, and they highlight the importance of using multidimensional measures of neighborhood conditions in public health research.

## Teaser

This cohort study assesses the association between long-term neighborhood economic trajectories and changes in weight status among Black and White participants in the Southern Community Cohort Study living in predominantly low-income communities in the southeastern US.

## Introduction

Obesity is not merely determined by genetic factors and personal choices; it is also associated with contextual factors.^1,2^ An increasing body of evidence has revealed that low socioeconomic status (SES) is associated with a higher prevalence and risk of obesity and weight gain among residents.^1^ Living in a low-income neighborhood has also been associated with substantial weight loss, which is a specific health risk factor for older adults.^3^ Neighborhoods are not static, and neighborhood change may have a distinct role in health.^4^ However, a recent scoping review^2^ of longitudinal studies found that few studies examined neighborhood change and weight outcomes, suggesting a need for more investigations.

Some previous studies,^5,6,7,8^ although not all,^9,10^ have found that neighborhood decline in SES was associated with obesity and excessive weight gain and loss, whereas improvement in neighborhood SES was associated with more favorable weight outcomes. A limitation of these earlier studies^5,6,7,8,9,10^ was the use of simplistic measures (eg, differences in poverty rates or a composite neighborhood SES index between 2 time points), which did not distinguish among specific types of neighborhood change. For example, improvement in the neighborhood SES index can be a consequence of overall economic growth without substantial residential relocation or of housing development that attracts individuals and families with higher income at the cost of displacing the original residents with lower income (ie, gentrification). It is important to make such distinctions because different types of neighborhood change may have different health consequences.

In the past 2 decades, there has been increasing research attention on the potential health consequences of neighborhood gentrification. Two recent systematic reviews^11,12^ reported inconsistent definitions of gentrification, mixed and inconclusive findings, and a substantial overall gap in understanding of the health implications of gentrification. Although gentrification has become a focal point of neighborhood research in public health, it is only 1 form of neighborhood change, and there has been little research on health consequences of other types of neighborhood change, such as overall growth without displacement or different forms of neighborhood decline.

A new method has been recently developed that uses simple and widely available national statistics to define multiple types of neighborhood trajectories in the US, including stability, growth, poverty concentration, abandonment, and gentrification or displacement.^13^ In the current cohort study, we used this new method^13^ to assess the association of long-term exposure to different neighborhood economic trajectories with weight gain and weight loss among participants in the ongoing Southern Community Cohort Study (SCCS), which enrolled a large cohort of Black and White adults residing in communities with relatively low SES. We restricted our analysis to Black and White populations in the SCCS because other racial and ethnic groups combined constituted less than 5% of the total baseline sample. We also investigated whether and in what ways the association between neighborhood economic trajectories and changes in weight status differed by race and sex.

## Methods

### Study Population

The SCCS is an ongoing prospective cohort study focused on health disparities in the US.^14^ During the baseline period (March 2002 to September 2009), approximately 85 000 men and women living in 12 states (Alabama, Arkansas, Florida, Georgia, Kentucky, Louisiana, Mississippi, North Carolina, South Carolina, Tennessee, Virginia, and West Virginia) enrolled in the study, with 86% of participants recruited from community health centers that primarily served uninsured and underinsured populations. The baseline survey collected information on sociodemographic characteristics, lifestyle and health characteristics, weight status, and residential addresses. From November 2008 to January 2013, a follow-up survey was conducted to collect updated information, including residential addresses and weight. The SCCS was approved by the institutional review boards of the Vanderbilt University Medical Center and Meharry Medical College. All participants in the SCCS provided written informed consent, which included consent for the use of the data in future studies. This study followed the Strengthening the Reporting of Observational Studies in Epidemiology (STROBE) reporting guideline for cohort studies.

The present study measured neighborhood economic trajectories of participants in the SCCS between 2000 and 2016. Data were analyzed from December 12, 2021, to July 16, 2022.

### Neighborhood Economic Trajectories

Methods used to define neighborhood economic trajectories were reported previously.^13^ In brief, we defined individuals with low income residing in a neighborhood between 2000 and 2016 as those who had incomes lower than 200% of the federal poverty line in each respective year. Next, we classified the 2000 to 2016 economic trajectory for a Census tract based on 2 measures: (1) changes in the absolute number of individuals without low income, which indicated changes in the desirability of an area, and (2) changes in the proportion of individuals with low income, which indicated the overall economic pattern occurring in the area during this period. A tract was classified as economically expanding if the population without low income increased by more than 10% and the proportion of the population with low income decreased by more than 5 percentage points. Likewise, a tract was classified as economically declining if the population without low income decreased by more than 10% and the proportion of the population with low income increased by more than 5 percentage points.

The second layer of categorization was based on changes in the size of the low-income population between 2000 and 2016. Tracts that were economically expanding were categorized in the growth trajectory group if their low-income population increased or in the displacement trajectory group if their low-income population decreased over this period. Tracts that were economically declining were categorized in the poverty concentration trajectory group if their low-income population increased or in the abandonment trajectory group if their low-income population decreased over this period. Tracts that were neither economically expanding nor declining were categorized in the stability trajectory group.

### Weight Change

Substantial weight gain or loss was defined as gaining or losing 10% or more of baseline weight. This cutoff was chosen because previous research found that a 10% change in weight was associated with increased mortality rates in middle-aged and older populations^15^ and that neighborhood socioeconomic deprivation was associated with weight outcomes in middle-aged and older adults in the US.^3,6^

### Covariates

The baseline questionnaire collected self-reported demographic characteristics (eg, race, sex, age, educational level, annual household income, household size, and marital status) and lifestyle factors (eg, physical activity, sleep, alcohol consumption, smoking, and dietary intake). We derived tract-level population density and poverty rates using US Census data from 2000, and we measured rural-urban status using rural-urban commuting area primary codes (which comprise 10 codes that classify US Census tracts using measures of population density, urbanization, and daily commuting, with code 1 indicating metropolitan area core; code 2, metropolitan area high commuting; code 3, metropolitan area low commuting; code 4, micropolitan area core; code 5, micropolitan high commuting; code 6, micropolitan area low commuting; code 7, small-town core; code 8, small-town high commuting; code 9, small-town low commuting; and code 10, rural area)^16^ from 2000.

### Analytic Sample

**Figure 1.**
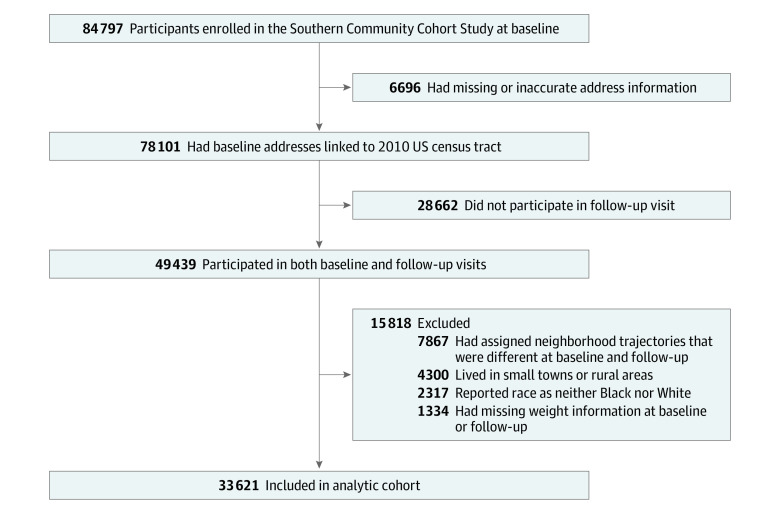
Study Flowchart

Among 84 797 participants enrolled in the SCCS at baseline, 78 101 had baseline addresses that were successfully matched to a point location and linked to a 2010 Census tract identification number. Of those, 49 439 individuals participated in the follow-up visit. We excluded 1334 participants who had missing data on weight status either at the baseline or follow-up visit and 2317 participants who reported their race as neither Black nor White. Because our focus was on long-term neighborhood exposure, we further excluded 7867 participants who had assigned neighborhood trajectories that were different between baseline and follow-up periods (eg, stability trajectory at baseline and growth trajectory at follow-up). We also excluded 4300 participants living in rural areas (n = 1591) or small towns (n = 2709) because the neighborhood change variable was developed for urban areas. The final analytic sample included 33 621 participants (28.2% of the total SCCS sample at baseline) (Figure 1). Differences in characteristics of participants who were included vs excluded are shown in the eTable in the Supplement.

### Statistical Analysis

We used a multinomial logistic regression analysis to calculate odds ratios (ORs) and 95% CIs to assess the association between neighborhood trajectory groups (poverty concentration, abandonment, displacement, growth, and stability [reference group]) and weight outcomes (gaining ≥10% of baseline weight, losing ≥10% of baseline weight, and weight change from baseline <10% [reference outcome]). We reported results from the base model, which was adjusted for age, sex, and race; the full model was further adjusted for educational level, household income and size, marital status, employment status, rural-urban commuting area, and tract-level population density and poverty rate (from 2000). We conducted an analysis of the overall sample and separate analyses of Black participants, White participants, women, and men. We defined statistical significance as nonoverlap between the 95% CI and 1. All analyses were performed using SAS software, version 9.4 (SAS Institute Inc).

## Results

Among 33 621 participants, the mean (SD) age was 53.4 (8.8) years; 22 116 participants (65.8%) were female, 11 505 (34.2%) were male, 21 782 (64.8%) were Black, and 11 839 (35.2%) were White. Overall, 19 246 individuals (57.2%) were residents of neighborhoods in the stability group, followed by the poverty concentration group (9483 individuals [28.2%]), the abandonment group (2778 individuals [8.3%]), the displacement group (1353 individuals [4.0%]), and the growth group (761 individuals [2.3%]) (Table 1).

**Table 1.** Baseline Characteristics of Participants in the Southern Community Cohort Study by Neighborhood Economic Trajectory Group^a^

Characteristic	Participants, No. (%)				
	Poverty concentration group (n = 9483)	Abandonment group (n = 2778)	Displacement group (n = 1353)	Growth group (n = 761)	Stability group (n = 19 246)
Age, mean (SD), y	53.2 (8.8)	52.9 (8.6)	53.9 (8.9)	53.2 (8.9)	53.6 (8.9)
Race					
Black	6271 (66.1)	2500 (90.0)	844 (62.4)	555 (72.9)	11 612 (60.3)
White	3212 (33.9)	278 (10.0)	509 (37.6)	206 (27.1)	7634 (39.7)
Sex					
Female	6367 (67.1)	1803 (64.9)	857 (63.3)	524 (68.9)	12 565 (65.3)
Male	3116 (32.9)	975 (35.1)	496 (36.7)	237 (31.1)	6681 (34.7)
Educational level less than high school	2116 (22.3)	919 (33.1)	337 (24.9)	186 (24.4)	4369 (22.7)
Married	3639 (38.4)	825 (29.7)	513 (37.9)	375 (49.3)	8238 (42.8)
Annual household income ≥$50 000	1149 (12.1)	115 (4.1)	180 (13.3)	120 (15.8)	3280 (17.0)
Unemployed	5302 (55.9)	1704 (61.3)	812 (60.0)	383 (50.3)	10 491 (54.5)
Current smoker	3247 (34.2)	1047 (37.7)	472 (34.9)	217 (28.5)	5855 (30.4)
Total physical activity, mean (SD), metabolic equivalent h/d	21.9 (17.7)	20.9 (17.5)	21.8 (18.2)	22.6 (17.1)	21.8 (17.3)
Time spent sitting, mean (SD), h/d	9.7 (5.1)	9.3 (5.0)	9.3 (4.9)	9.0 (4.7)	9.4 (4.9)
Alcohol consumption ≥1 drink/d	1594 (16.8)	573 (20.6)	297 (22.0)	120 (15.8)	3305 (17.2)
Healthy eating index score, mean (SD)^b^	59.4 (12.4)	58.1 (12.0)	59.3 (11.9)	59.6 (11.9)	59.7 (12.2)
RUCA primary code in 2000^c^					
Metropolitan area					
Core (code 1)	7758 (81.8)	1976 (71.1)	790 (58.4)	238 (31.3)	11 779 (61.2)
Commuting (code 2 or 3)	501 (5.3)	35 (1.3)	333 (24.6)	493 (64.8)	3652 (19.0)
Micropolitan area					
Core (code 4)	963 (10.2)	672 (24.2)	121 (8.9)	22 (2.9)	2220 (11.5)
Commuting (code 5 or 6)	261 (2.8)	95 (3.4)	109 (8.1)	8 (1.1)	1594 (8.3)
Population density within tract in 2000, median (IQR), people/km^2^	896 (359-1423)	1209 (587-1824)	596 (28-1556)	21 (11-144)	405 (60-1025)
Poverty rate within tract in 2000, mean (SD), %	20.3 (10.7)	36.0 (11.5)	26.5 (14.3)	19.1 (11.0)	22.5 (16.2)

^a^ Neighborhood economic trajectories were measured from 2000 to 2016. The baseline period for participants was from March 2002 to September 2009.

^b^ Score range, 0 to 100, with higher scores indicating a diet that better aligns with dietary recommendations from the US Department of Agriculture.

^c^ Rural-urban commuting area (RUCA) primary codes classify US Census tracts using measures of population density, urbanization, and daily commuting. Codes range from 1 to 10, with code 1 indicating metropolitan area core; code 2, metropolitan area high commuting; code 3, metropolitan area low commuting; code 4, micropolitan area core; code 5, micropolitan high commuting; code 6, micropolitan area low commuting; code 7, small-town core; code 8, small-town high commuting; code 9, small-town low commuting; and code 10, rural area.

In the stability (reference) group, 11 612 participants (60.3%) were Black, 7634 (39.7%) were White, 12 565 (65.3%) were female, and 6681 (34.7%) were male. Overall, 4369 participants (22.7%) in the stability group did not complete high school, 8238 (42.8%) were married, 3280 (17.0%) had annual household income of $50 000 or greater, 10 491 (54.5%) were unemployed, 5855 (30.4%) reported current smoking, and 3305 (17.2%) reported alcohol consumption of 1 or more drinks per day. A total of 11 779 participants (61.2%) in the stability group lived in metropolitan core areas, and 3652 (19.0%) lived in metropolitan commuting areas, with a median (IQR) population density of 405 (60-1025) people/km^2^ and a mean (SD) poverty rate of 22.5% (16.2%).

Compared with residents in the stability group, those in the abandonment group had the most distinct profile. Participants living in abandonment tracts were more likely to be Black (2500 individuals [90.0%]) and unemployed (1704 individuals [61.3%]) and to report current smoking (1047 individuals [37.7%]) and alcohol consumption of 1 or more drinks per day (573 individuals [20.6%]). Participants in the abandonment vs stability group were less likely to have completed high school (919 individuals [33.1%]), be married (825 individuals [29.7%]), or report a household income of $50 000 or greater (115 individuals [4.1%]). Compared with those in stability tracts, participants in abandonment tracts were more likely to live in metropolitan core areas (1976 individuals [71.1%]) with higher population density (median [IQR], 1209 [587-1824] people/km^2^) and a higher poverty rate (mean [SD], 36.0% [11.5%]).

Other neighborhood trajectory groups had several distinct features. For example, relative to the stability group, the poverty concentration group had lower annual household income (≥$50 000: 1149 individuals [12.1%]), a higher proportion of participants living in metropolitan core areas (7758 individuals [81.8%]), and higher population density (median [IQR], 896 [359-1423] people/km^2^), whereas the growth group had a higher proportion of Black participants (555 individuals [72.9%]), a greater proportion of participants living in metropolitan commuting areas (493 individuals [64.8%]), and the lowest population density at baseline (median [IQR], 21 [11-144] people/km^2^). Participant and neighborhood characteristics in the displacement group were most comparable with those in the stability group (eg, 844 individuals [62.4%] were Black, 857 [63.3%] were female, 337 [24.9%] did not complete high school, and 790 [58.4%] lived in a metropolitan core area with a median [IQR] population density of 596 [28-1556] people/km^2^).

**Figure 2.**
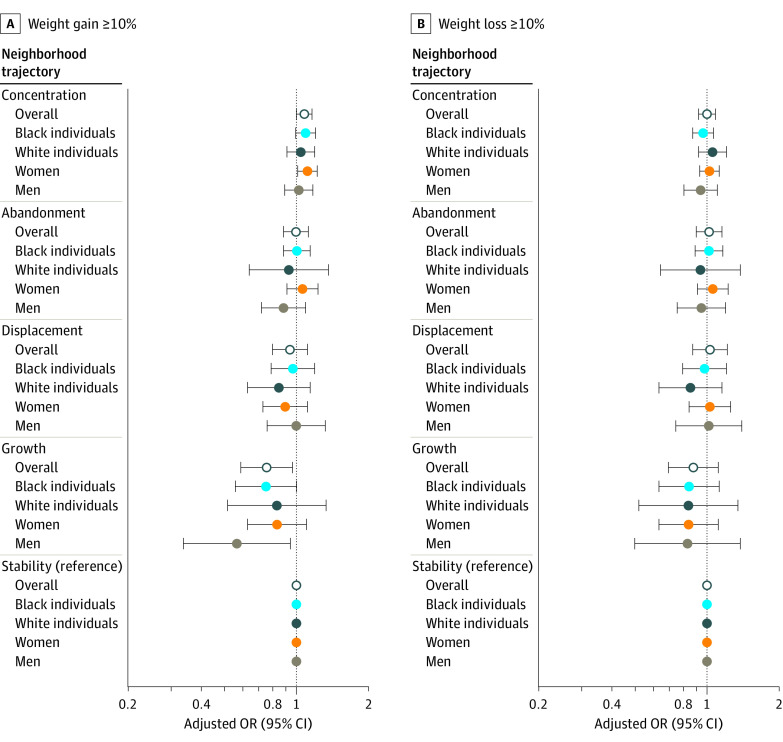
Association of Neighborhood Trajectories With Weight Gain and Weight Loss During Baseline and Follow-up Periods in Overall Sample and by Race and Sex The baseline period for participants was from March 2002 to September 2009, and the follow-up period was from November 2008 to January 2013. Models were adjusted for age (continuous), sex (male or female), race (Black or White), educational level (less than high school, high school graduate, some college, college graduate, or missing), annual household income (≤$15 000, $15 999 to <$25 000, $25 000 to <$50 000, ≥$50 000, or missing), employment status (employed, unemployed, or missing), rural-urban commuting area primary code (metropolitan core, metropolitan commuting, micropolitan core, or micropolitan commuting), population density (quintiles), and poverty rate (continuous) at the Census tract level. OR indicates odds ratio.

The associations between neighborhood trajectories and weight gain and loss are shown in Table 2 and Figure 2. In the minimally adjusted model, compared with the stability group, the poverty concentration group was associated with a significantly higher likelihood of gaining 10% or more of baseline weight (OR, 1.11; 95% CI, 1.04-1.20), whereas the growth group was associated with a significantly lower likelihood of substantial weight gain (OR, 0.70; 95% CI, 0.55-0.89). These findings were modestly attenuated after adjustment for confounders in the full model but remained statistically significant (poverty concentration group: OR, 1.08 [95% CI, 1.00-1.17]; growth group: OR, 0.75 [95% CI, 0.58-0.97]). None of the neighborhood trajectories had a significant association with weight loss of 10% or more compared with the stability group in the fully adjusted model (poverty concentration group: OR, 1.00 [95% CI, 0.93-1.09]; abandonment group: OR, 1.01 [95% CI, 0.84-1.15]; displacement group: OR, 1.04 [95% CI, 0.83-1.23]; growth group: OR, 0.88 [95% CI, 0.69-1.12]).

**Table 2.** Association Between Neighborhood Economic Trajectories and Weight Gain or Weight Loss During Baseline and Follow-up Periods in Overall Sample^a^

Outcome	Trajectory group, OR (95% CI)				
	Poverty concentration (n = 9483)	Abandonment (n = 2778)	Displacement (n = 1353)	Growth (n = 761)	Stability (n = 19 246)
**Weight gain ≥10%**					
Participants, No. (%)	1341 (14.1)	392 (14.1)	166 (12.3)	74 (9.7)	2444 (12.7)
Base model^b^	1.11 (1.04-1.20)	1.11 (0.98-1.25)	0.99 (0.83-1.17)	0.70 (0.55-0.89)	1 [Reference]
Full model^c^	1.08 (1.00-1.17)	0.97 (0.86-1.10)	0.93 (0.78-1.11)	0.75 (0.58-0.97)	1 [Reference]
**Weight loss ≥10%**					
Participants, No. (%)	1161 (12.2)	374 (13.5)	173 (12.8)	83 (10.9)	2326 (12.1)
Base model^b^	1.02 (0.95-1.10)	1.13 (1.00-1.27)	1.07 (0.90-1.26)	0.83 (0.66-1.05)	1 [Reference]
Full model^c^	1.00 (0.93-1.09)	1.01 (0.89-1.15)	1.04 (0.83-1.23)	0.88 (0.69-1.12)	1 [Reference]

Abbreviation: OR, odds ratio.

^a^ Neighborhood economic trajectories were measured from 2000 to 2016. The baseline period for participants was from 2002 to September 2009. The follow-up period was from November 2008 to January 2013.

^b^ Adjusted for age (continuous), sex (male or female), and race (Black or White).

^c^ Adjusted for educational level (less than high school, high school graduate, some college, college graduate, or missing), annual household income (≤$15 000, $15 999 to <$25 000, $25 000 to <$50 000, ≥$50 000, or missing), household size (0, 1, 2, 3, or >3 residents), employment status (employed, unemployed, or missing), rural-urban commuting area primary code (metropolitan core, metropolitan commuting, micropolitan core, or micropolitan commuting), population density (quintiles), and poverty rate (continuous) at the Census tract level.

Sex-specific and race-specific results are shown in Table 3 and Figure 2. In the full model, a significant positive association between the poverty concentration group and substantial weight gain was observed among women (OR, 1.12; 95% CI, 1.02-1.23) but not men (OR, 1.02; 95% CI, 0.88-1.17) and among Black participants (OR, 1.10; 95% CI, 1.00-1.22) but not White participants (OR, 1.03; 95% CI, 0.90-1.18). However, in the growth group, a significant negative association with substantial weight gain was found among men (OR, 0.58; 95% CI, 0.35-0.96) but not women (OR, 0.83; 95% CI, 0.62-1.12), and no significant association was observed among Black participants (OR, 0.76; 95% CI, 0.56-1.02) or White participants (OR, 0.84; 95% CI, 0.52-1.36). The interaction terms between neighborhood trajectories and both race (*P* = .78) and sex (*P* = .70) were not statistically significant.

**Table 3.** Association Between Neighborhood Economic Trajectories and Weight Gain or Weight Loss During Baseline and Follow-up Periods by Race and Sex^a^

Outcome	Trajectory group, OR (95% CI)				
	Poverty concentration	Abandonment	Displacement	Growth	Stability
**Black adults**					
Weight gain ≥10%					
Participants, No./total No. (%)	919/6271 (14.7)	357/2500 (14.3)	114/844 (13.5)	54/555 (9.7)	1552/11 612 (13.4)
Base model^b^	1.10 (1.01-1.21)	1.11 (0.97-1.25)	1.05 (0.85-1.30)	0.66 (0.49-0.88)	1 [Reference]
Full model^c^	1.10 (1.00-1.22)	0.98 (0.86-1.12)	0.97 (0.78-1.19)	0.76 (0.56-1.02)	1 [Reference]
Weight loss ≥10%					
Participants, No./total No. (%)	755/6271 (12.0)	338/2500 (13.5)	117/844 (13.9)	61/555 (11.0)	1476/11 612 (12.7)
Base model^b^	0.95 (0.86-1.04)	1.10 (0.96-1.25)	1.12 (0.91-1.38)	0.79 (0.60-1.04)	1 [Reference]
Full model^c^	0.96 (0.87-1.07)	1.01 (0.88-1.15)	1.09 (0.88-1.33)	0.85 (0.64-1.13)	1 [Reference]
**White adults**					
Weight gain ≥10%					
Participants, No./total No. (%)	422/3212 (13.1)	35/278 (12.6)	52/509 (10.2)	20/206 (9.7)	892/7634 (11.7)
Base model^b^	1.13 (0.99-1.28)	1.11 (0.77-1.61)	0.87 (0.65-1.18)	0.83 (0.51-1.33)	1 [Reference]
Full model^c^	1.03 (0.90-1.18)	0.87 (0.59-1.28)	0.83 (0.61-1.13)	0.84 (0.52-1.36)	1 [Reference]
Weight loss ≥10%					
Participants, No./total No. (%)	406/3212 (12.6)	36/278 (12.9)	56/509 (11.0)	22/206 (10.7)	850/7634 (11.1)
Base model^b^	1.16 (1.02-1.32)	1.21 (0.84-1.73)	0.96 (0.72-1.29)	0.93 (0.59-1.47)	1 [Reference]
Full model^c^	1.05 (0.91-1.20)	0.99 (0.68-1.45)	0.97 (0.72-1.30)	0.97 (0.61-1.54)	1 [Reference]
**Women**					
Weight gain ≥10%					
Participants, No./total No. (%)	958/6367 (15.0)	269/1803 (14.9)	103/857 (12.0)	57/524 (10.9)	1678/12 566 (13.4)
Base model^b^	1.16 (1.06-1.26)	1.15 (1.00-1.33)	0.92 (0.74-1.15)	0.76 (0.57-1.01)	1 [Reference]
Full model^c^	1.12 (1.02-1.23)	1.04 (0.90-1.21)	0.89 (0.72-1.11)	0.83 (0.62-1.12)	1 [Reference]
Weight loss ≥10%					
Participants, No./total No. (%)	888/6367 (13.9)	271/1803 (15.0)	125/857 (14.6)	65/524 (12.4)	1721/12 566 (13.7)
Base model^b^	1.04 (0.96-1.14)	1.13 (0.98-1.30)	1.05 (0.86-1.29)	0.86 (0.66-1.12)	1 [Reference]
Full model^c^	1.04 (0.95-1.14)	1.05 (0.91-1.22)	1.04 (0.85-1.28)	0.89 (0.68-1.17)	1 [Reference]
**Men**					
Weight gain ≥10%					
Participants, No./total No. (%)	383/3116 (12.3)	123/975 (12.6)	63/496 (12.7)	17/237 (7.2)	766/6681 (11.5)
Base model^b^	1.03 (0.90-1.18)	1.03 (0.83-1.27)	1.11 (0.85-1.48)	0.56 (0.34-0.92)	1 [Reference]
Full model^c^	1.02 (0.88-1.17)	0.84 (0.68-1.05)	0.98 (0.74-1.30)	0.58 (0.35-0.96)	1 [Reference]
Weight loss ≥10%					
Participants, No./total No. (%)	273/3116 (8.8)	103/975 (10.6)	48/496 (9.7)	18/237 (7.6)	605/6681 (9.1)
Base model^b^	0.96 (0.82-1.11)	1.12 (0.89-1.40)	1.08 (0.79-1.48)	0.76 (0.47-1.25)	1 [Reference]
Full model^c^	0.92 (0.78-1.07)	0.91 (0.72-1.15)	1.03 (0.75-1.41)	0.85 (0.52-1.41)	1 [Reference]

Abbreviation: OR, odds ratio.

^a^ Neighborhood economic trajectories were measured from 2000 to 2016. The baseline period for participants was from March 2002 to September 2009. The follow-up period was from November 2008 to January 2013.

^b^ Adjusted for age (continuous), sex (male or female), and race (Black or White).

^c^ Adjusted for educational level (less than high school, high school graduate, some college, college graduate, or missing), annual household income (≤$15 000, $15 999 to <$25 000, $25 000 to <$50 000, ≥$50 000, or missing), household size (0, 1, 2, 3, or >3 residents), employment status (employed, unemployed, or missing), rural-urban commuting area primary code (metropolitan core, metropolitan commuting, micropolitan core, or micropolitan commuting), population density (quintiles), and poverty rate (continuous) at the Census tract level.

## Discussion

In this cohort study of Black and White adults from primarily disadvantaged communities in the southeastern US, different types of neighborhood economic change were associated with weight gain. Compared with residents in economically stable neighborhoods, those in neighborhoods with an economic growth trajectory were less likely to experience substantial weight gain, whereas those in neighborhoods with a poverty concentration trajectory were more likely to experience substantial weight gain. We found potential race and sex differences, but the effect modification based on race and sex was not statistically significant. In contrast, neighborhood economic trajectories were not associated with substantial weight loss over the follow-up period.

Several prospective studies^5,6^ have reported an association between neighborhood change and weight outcomes. For example, the Dallas Heart Study^5^ found that improvement in neighborhood SES was associated with lower weight gain. In addition, a recent analysis of data from the National Institutes of Health–American Association of Retired Persons Health and Diet Study^6^ found that every 5-percentile increase in the national ranking of neighborhood SES was associated with a 1.2% to 2.4% decrease in the odds of excessive weight gain or loss. However, by assessing change using a single neighborhood SES measure, these earlier studies^5,6^ were not able to examine more refined categories of neighborhood change, which was an important research gap that our analysis aimed to address. Our approach to measuring neighborhood change was to first define neighborhoods based on economic trajectories (ie, expanding vs declining), consistent with the neighborhood improvement and decline trajectories used in previous research.^5,6^ However, unlike earlier studies,^5,6^ we further separated each trajectory (expanding vs declining) into 2 subcategories according to changes in the size of the low-income population, which provided additional information about the relocation patterns of residents with low income (eg, remaining in vs leaving neighborhoods that were economically expanding or declining).

Notably, our findings revealed that subcategories within the same economic trajectory may have differential associations with weight outcomes. Both the growth and displacement trajectory groups were characterized as economically expanding, but only the former was associated with a lower likelihood of substantial weight gain. A higher likelihood was observed only among residents in the poverty concentration group but not the abandonment group, although both trajectory types were characterized as economically declining. These differential patterns highlighted the limitation of a single measure–based assessment of neighborhood change.

Although neighborhood economic improvement is generally viewed as a positive change, there has been controversy regarding the consequences of neighborhood gentrification, which occurs when low-income urban neighborhoods undergo considerable housing development leading to an influx of more affluent residents.^17,18^ Gentrification can revitalize low-income neighborhoods by bringing in employment opportunities and material resources. In particular, changes in physical activity and food environment associated with decreases in crime and increases in green space, sidewalks, and outlets for healthy foods^12^ may encourage healthier lifestyles and prevent weight gain.^19^ However, gentrification can lead to displacement of the original residents, disruption of neighborhood social networks, and increases in the cost of living, which may create a more stressful environment that cultivates less healthy behaviors resulting in weight gain and obesity, particularly among individuals and families at the poverty level.^20^ We found no association between displacement neighborhoods and weight outcomes, suggesting that health benefits conferred by neighborhood economic growth may be offset by other detrimental consequences of gentrifying neighborhoods. Notably, the participants in our study may have been especially susceptible to the adverse effects of neighborhood gentrification because of their relatively low SES. It is also worth noting that gentrification can displace residents with low income into neighborhoods with lower SES and thus may have direct implications for poverty concentration, which was associated with substantial weight gain in our study. This possibility suggests that those who relocate to neighborhoods with low income due to gentrification may develop adverse weight outcomes. In contrast, the likelihood of developing substantial weight gain was significantly lower among residents of neighborhoods with growth trajectories, suggesting that economic development without displacement may confer health benefits.

Previous studies have generally found associations between neighborhood economic decline and adverse health outcomes, including higher weight gain,^3,5,6^ preterm birth rates,^21^ colorectal cancer incidence,^22^ and all-cause and cardiovascular mortality rates.^21^ However, research focusing on different types of neighborhood decline has been limited. To our knowledge, the present study was the first to separate declining neighborhoods into poverty concentration and abandonment trajectory types, each representing a different set of mechanisms underlying neighborhood decline. The abandonment type was relatively rare and typically confined to city centers with comprehensive decay. However, the poverty concentration type was generally found in aging cities and suburbs with lagging economic development. A previous analysis^13^ of the 50 largest metropolitan areas found that poverty concentration was the most common form of neighborhood change, affecting 36 million people in 2016. In our analysis of participants in the SCCS, 28.2% of the study sample lived in neighborhoods with poverty concentration trajectories between 2000 and 2016. The large size of the population living in these neighborhoods combined with the higher likelihood of weight gain observed in our study suggests a need for future studies to fully understand the health risks among residents in areas with poverty concentration.

Several studies^3,6,23,24^ have examined the race-specific and sex-specific associations between neighborhood environment and weight outcomes. An analysis of the California Health Interview Survey^23^ found that each 10% increase in the proportion of adults with a high school educational level or less was associated with a 14% increase in the odds of obesity among Black individuals (OR, 1.14; 95% CI, 0.96-1.36) and a 27% increase in the odds of obesity among non-Hispanic White individuals (OR, 1.27; 95% CI, 1.21%-1.33%). In another study,^24^ the association between neighborhood and obesity appeared to be similar across different racial, ethnic, and sex groups. In a large prospective study,^3^ lower neighborhood SES was associated with higher weight gain in both men and women, although the pattern appeared stronger among men. However, in another analysis involving the same cohort,^6^ the association between 20-year neighborhood trajectories and weight outcomes was similar between sexes. More studies are needed to clarify the association between neighborhood and weight outcomes in subpopulations with different sociodemographic characteristics.

### Strengths and Limitations

This study has several strengths. First, our measurement of neighborhood change was novel, and some of the neighborhood trajectory types, such as growth, poverty concentration, and abandonment, have rarely been examined. Second, our analysis was conducted among a diverse cohort, allowing for race-specific and sex-specific investigation. Third, we studied not only weight gain but weight loss, which is an often overlooked factor associated with health.^15^ The lack of association between neighborhood change and weight loss in the study sample may be explained by the relatively young ages of participants in the SCCS compared with other cohorts in which significant results were reported.^3,6^ The population of our study was also distinct because most participants were recruited from low-income communities, which have been underexamined in previous research.

This study also has limitations. First, only a small proportion of participants lived in neighborhoods with growth and abandonment trajectories, and the characteristics of these neighborhoods were distinct. The finding of differences in individual and neighborhood characteristics across different neighborhood trajectories suggests that factors other than neighborhood change may confound the association, which is an innate limitation of observational studies. Second, our measure of neighborhood change was primarily developed for larger urban areas and cannot be applied to small towns and rural areas, which have also undergone considerable change in past decades. More studies are needed to understand the consequences of neighborhood change for these communities. Third, weight was self-reported and subject to error and bias. Fourth, our analytic sample included only 39.6% of the original cohort, mainly because of missing data on important exposure and outcome variables, unavailability for follow-up, and differences among those who were included and excluded (eTable in the Supplement); these limitations suggest that findings cannot be generalized to the whole study population. Fifth, we focused on participants who lived in the same type of neighborhood during baseline and follow-up periods. Due to the small sample, we were not able to examine the association between relocation to neighborhoods with different trajectories and weight outcomes, which is an important research question that warrants further investigation.

## Conclusions

In this longitudinal cohort study of Black and White adults in the US, neighborhood economic growth without displacement was associated with lower odds of substantial weight gain, whereas poverty concentration was associated with higher odds of weight gain. Although this observational study could not establish causal relationships, the findings suggest that neighborhood trajectories are important factors associated with weight outcomes. The study’s results also highlight the importance of using more nuanced and multidimensional measures of neighborhood change in public health research.

## Notes

Supplement. **eTable.** Baseline Participant Characteristics According to Inclusion and Exclusion Status
