# Supplementary material for: Association of Neighborhood Economic Trajectories With Changes in Weight Status Among Black and White Adults in the Southeastern US
Source: JAMA Netw Open. 2022 Sep 8;5(9):e2230697. doi: 10.1001/jamanetworkopen.2022.30697 (PMC9459659; doi:10.1001/jamanetworkopen.2022.30697)
Supplement: Supplement. — eTable. Baseline Participant Characteristics According to Inclusion and Exclusion Status [file jamanetwopen-e2230697-s001.pdf]

Supplementary Online Content

Xiao Q, Myott E, Schlundt DG, Stancil W. Association of neighborhood economic trajectories with changes in weight status among Black and White adults in the southeastern US. *JAMA Netw Open*. 2022;5(9):e2230697. doi:10.1001/jamanetworkopen.2022.30697

eTable. Baseline Participant Characteristics According to Inclusion and Exclusion Status

This supplementary material has been provided by the authors to give readers additional information about their work.

© 2022 Xiao Q et al. *JAMA Network Open.*

eTable. Baseline Participant Characteristics According to Inclusion and Exclusion Status

Study characteristics, N (%)	Excluded	Included
Age, year, mean (SD)	53.4 (8.8)	51.5 (8.7)
Black	32155 (66.2)	21782 (64.8)
Female	26960 (54.9)	22116 (65.8)
Education, less than high school	15576 (31.7)	7927 (23.6)
Married	15451 (31.5)	13590 (40.4)
Household income >50k	3138 (6.4)	4844 (14.4)
Unemployed	30104 (61.3)	18692 (55.6)
Current smoker	22601 (46.0)	10838 (32.2)
Total physical activity, MET-hour/day, mean (SD)	21.7 (17.5)	23.4 (20.1)
Sitting, hour/day, mean (SD)	9.5 (5.0)	9.2 (5.1)
Alcohol consumption, 1+ drink/day	11326 (23.1)	5889 (17.5)
Health eating index, mean (SD)	59.5 (12.2)	56.7 (11.8)
Population density within tract (2000), per km^2, mean (SD)	818.4 (837.3)	848.6 (934.5)
Poverty rate within tract (2000), mean (SD)	23.1 (14.9)	26.7 (14.6)

© 2022 Xiao Q et al. *JAMA Network Open.*
